# Factors Associated With Parents’ Intent to Vaccinate Adolescents for Human Papillomavirus: Findings From the 2014 National Immunization Survey–Teen

**DOI:** 10.5888/pcd14.160314

**Published:** 2017-06-08

**Authors:** Kahee A. Mohammed, Elaina Vivian, Travis M. Loux, Lauren D. Arnold

**Affiliations:** 1 Saint Louis University Center for Outcomes Research, St Louis, Missouri; 2 Department of Epidemiology, College for Public Health and Social Justice, Saint Louis University, St Louis, Missouri; 3 Department of Internal Medicine, School of Medicine, Saint Louis University, St Louis, Missouri; 4 Department of Health Management and Policy, College for Public Health and Social Justice, Saint Louis University, St Louis, Missouri; 5 Methodist Dallas Medical Center, Dallas, Texas; 6 Department of Biostatistics, College for Public Health and Social Justice, Saint Louis University, St Louis, Missouri

## Abstract

**Introduction:**

While factors associated with receipt of human papillomavirus (HPV) vaccination have been well characterized, less is known about the characteristics associated with parents’ intent to have their adolescent children vaccinated. This study aimed to examine factors associated with parental intention toward HPV vaccination.

**Methods:**

We analyzed data on 10,354 adolescents aged 13 to 17 years from the 2014 National Immunization Survey–Teen. Weighted multivariable logistic regression was used to examine associations between sociodemographic characteristics of mothers and adolescents, as well as a health care provider recommendation with parents’ intention to have their children receive HPV vaccine.

**Results:**

Among unvaccinated adolescents, Hispanic ethnicity (boys adjusted odds ratio [AOR], 1.87, 95% confidence interval [CI], 1.34–2.61; and girls AOR, 1.57; 95% CI, 1.05–2.35), mothers with less than a high school diploma (boys AOR, 2.41; 95% CI, 1.58–3.67; and girls AOR, 1.86; 95% CI, 1.02–3.38), and having a health care provider recommend the vaccine (boys AOR, 1.87; 95% CI, 1.52–2.31; and girls AOR, 1.38; 95% CI, 1.05–1.82) were significantly associated with parents’ intention to have their adolescent child vaccinated within the next 12 months. In addition, non-Hispanic black race was a significant predictor of parents’ intent to vaccinate for boys (AOR, 1.89; 95% CI, 1.35–2.65).

**Conclusion:**

Maternal education and Hispanic ethnicity were the strongest predictors of parental intent to vaccinate against HPV, followed by provider recommendation. As HPV vaccination rates in the United States remain below the Healthy People 2020 goal, messages may need to be targeted based on maternal education, race/ethnicity, and provider recommendation.

## Introduction

Nearly all sexually active adults in the United States will acquire human papillomavirus (HPV) infection during their lifetime (1). If infection with a high-risk HPV strain persists beyond 1 to 2 years, anogenital cancers (cervical, penile, and anal cancer) can result (2). Incidence of HPV-associated cancer is disproportionately higher among African Americans, Hispanics, and those living in poverty (3).

The Advisory Committee on Immunization Practices (ACIP) recommends routine HPV vaccination for US adolescents aged 11 and 12 years (4), with catch-up vaccination through age 26 (4). Despite a Healthy People 2020 goal of 80% vaccine coverage among US adolescents (5), the 2014 National Immunization Survey–Teen (NIS-Teen) survey found that only 60.0% of girls and 41.7% of boys aged 13 to 17 years received at least 1 dose of the vaccine (6). Moreover, only 39.7% of girls and 21.6% of boys completed the vaccine series (6); at the time data were collected, 3 doses of vaccine were required to complete the HPV vaccine series (7). This suboptimal uptake is attributed to a lack of physician recommendation, safety concerns, and inadequate knowledge about HPV (8–12). In the United States, the Patient Protection and Affordable Care Act now requires that all insurance plans cover the cost of ACIP-recommended vaccines, including HPV vaccine (13). The Vaccines for Children program provides HPV vaccination at no cost for underinsured and uninsured children younger than 19 years (14).

While factors associated with receipt of HPV vaccination are well characterized, less is known about characteristics associated with parental intent to have their children vaccinated against HPV. This understanding is necessary when considering progression across the stages of health behavior change, as described by the Transtheoretical Model (15). Intent — or preparation — occurs when a person makes the decision to engage in a behavior (eg, HPV vaccination) but has not yet taken action. Preparation follows contemplation, where the person considers the options (eg, HPV vaccination vs no HPV vaccination). Understanding factors associated with intent to have adolescents receive HPV vaccination can inform interventions designed to move parents through the stages of change, from considering HPV vaccination for their adolescents to intention to vaccinate to actual vaccination. To the best of our knowledge, no national studies have examined predictors of HPV vaccination intent. Thus, we used NIS-Teen 2014 data to examine factors associated with parental intention to vaccinate their adolescents against HPV.

## Methods

### Data source

The NIS-Teen is an annual cross-sectional survey that uses 2 phases of data collection to obtain vaccination information from a national probability sample of parents and guardians of US adolescents (16,17). In the first phase of data collection, a random-digit–dialed landline and cellular telephone survey is conducted to identify households with children aged 13 to 17 years. Parents provide information about their children’s sociodemographic characteristics, vaccination history, and health care providers. In the second phase, a Provider Record Check is sent to all identified providers to obtain provider-verified vaccination records for each respondent (17). The landline and the cellular telephone response rates for 2014 were 60.3% and 31.2%, respectively. Because the NIS-Teen 2014 data set is publicly available, the Saint Louis University institutional review board determined that this project did not constitute human subjects research.

### Measures

NIS-Teen 2014 captured vaccination information for 38,703 eligible adolescent boys and girls aged 13 to 17 years in United States. Of these, 21,057 adolescents (10,866 boys and 10,191 girls) had adequate provider-verified vaccination records. Our study was limited to parents of 10,354 adolescents (4,047 [39.7%] boys and 6,307 [60.3%] girls) with adequate provider-verified vaccination records who had not received any dose of HPV vaccine and whose parents were subsequently asked about their intention to have their child vaccinated against HPV in the 12 months following the survey.

The primary outcome of interest was intention to receive HPV vaccine, as ascertained from the survey question “How likely is it that teen will receive HPV shots in next 12 months?” Response options included “very likely,” “somewhat likely,” “not too likely,” “not likely at all,” and “not sure/don’t know.” For the purpose of this analysis, intention to receive HPV vaccine was dichotomized into “likely” and “not likely.” The 574 respondents (parents of 386 [7.6%] boys and 188 [6.1%] girls) who answered “not sure/don’t know” were excluded; however, a subanalysis of these parents (“not sure/don’t know” vs all other categories) was also conducted.

Adolescent sociodemographic covariates were sex, race/ethnicity (non-Hispanic white, non-Hispanic black, Hispanic, or non-Hispanic other [including multiple races/ethnicities]), and poverty status. Poverty status (above poverty or below poverty) was assessed by comparing respondents’ household income to the US Census poverty level (18). Because of known geographic variation in vaccine uptake (19), 4 census regions were used (Northeast, Midwest, South, or West). The adolescent’s mother’s sociodemographic covariates were maternal age (≤34 y, 35–44 y, or ≥45 y), education level (less than high school diploma, high school diploma or general equivalency degree, some college, or college graduate), marital status (married or never married), and number of children aged 18 years or younger (1, 2 or 3, or ≥4). Provider-level covariates were provider recommendation for HPV vaccination (yes or no) and number of valid, unique health care providers identified by respondents (1, 2, or ≥3).

### Statistical analysis

Differences in characteristics of adolescents were evaluated by using χ^2^ tests, stratified by the adolescent’s sex. Weighted multivariate binary logistic regression analyses were performed to examine the association between adolescents’ sociodemographic characteristics and the parents’ intention to have them receive HPV vaccine in the next 12 months, stratified by the adolescents’ sex. To test for differences in associations between boys and girls, 2-tailed *z* tests were performed by using the estimate and standard errors of the logistic regression coefficients (log odds ratios). This method is equivalent to testing for interactions within a single model but keeps the individual models more interpretable. A second multivariate logistic regression was conducted to examine factors associated with the parental answer, “not sure/don’t know” (about future HPV vaccination) versus all other categories of intent (ie, likely or unlikely to vaccinate). All analyses were performed in SAS version 9.4 (SAS Institute, Inc) and adjusted with weights as provided in the NIS-Teen 2014 data set to be representative of the US population. The level of statistical significance for all analyses was *P* < .05. To preserve sample size and reduce potential bias, missing values for explanatory variables were treated as a separate category in the logistic regression models but were not interpreted in the results.

## Results

Table 1 presents weighted demographic characteristics of adolescents aged 13 to 17 years who had not received any dose of HPV vaccine, stratified by sex. Adolescents were largely non-Hispanic whites living above the poverty level and residing in the South. Mothers were largely aged 45 years or older, married, and college graduates. Parents of 30.8% of boys and 53.1% of girls had received a health care provider recommendation for HPV vaccination for their child (*P* < .001). Approximately 7% of all survey respondents responded “not sure/don’t know” for whether they would vaccinate their adolescents for HPV in the next 12 months (data not shown).

**Table 1:** Characteristics of Adolescents Aged 13 to 17 Years Not Vaccinated Against Human Papillomavirus (HPV), by Sex, National Immunization Survey–Teen, 2014

Variable	Boys (n = 4,047), n (Weighted %)^a^	Girls (n = 6,307), n (Weighted %)^a^	*P* Value^b^
**Age, y**			
13	1,330 (21.9)	1,046 (25.1)	.03
14	1,297 (20.4)	913 (21.9)	
15	1,244 (18.0)	729 (18.9)	
16	1,290 (21.8)	711 (17.9)	
17	1,146 (18.0)	648 (16.1)	
**Race/ethnicity**			
Non-Hispanic white	4,446 (60.7)	2,737 (58.0)	.34
Non-Hispanic black	527 (12.7)	356 (12.0)	
Hispanic	784 (18.1)	522 (19.8)	
Non-Hispanic other^c^	550 (8.5)	432 (10.2)	
**Census region**			
South	2,329 (40.0)	1,458 (39.3)	.98
Midwest	1,452 (23.3)	920 (23.8)	
Northeast	1,015 (14.8)	661 (14.8)	
West	1,511 (22.0)	1,008 (22.1)	
**Poverty status^d^**			
Above poverty level	5,155 (74.4)	3,325 (76.6)	.27
Below poverty level	925 (21.0)	563 (23.4)	
Unknown	227 (4.6)	159 (3.5)	
**Mother’s education**			
Less than high school diploma	580 (11.3)	365 (12.3)	.87
High school diploma or general equivalency degree	1,101 (23.6)	722 (23.8)	
Some college	1,770 (26.9)	1,170 (26.9)	
College graduate	2,856 (38.1)	1,790 (37.0)	
**Mother’s marital status**			
Married	4,810 (68.8)	3,081 (70.6)	.34
Never married	1,497 (31.2)	966 (29.4)	
**Mother’s age, y**			
≤34	407 (7.0)	289 (8.1)	.23
35–44	2,585 (45.8)	1,640 (42.9)	
≥45	3,315 (47.2)	2,118 (50.0)	
**Number of mother’s children aged <18 y**			
1	2,519 (33.1)	1,543 (31.5)	.16
2 or 3	3,164 (54.4)	2,043 (53.5)	
≥4	624 (12.4)	461 (15.0)	
**Number of health care providers**			
1	3,233 (53.3)	2,048 (50.7)	.41
2	1,879 (28.3)	1,207 (29.7)	
≥3	1,177 (18.3)	785 (19.6)	
**Health care provider recommended HPV vaccine**			
Yes	2,146 (30.8)	2,230 (53.1)	<.001
No	4,061 (67.2)	1,740 (44.4)	
Missing	100 (1.9)	77 (2.4)	
**Parent intends to have child receive HPV vaccine in next 12 months**			
Not likely	3,343 (54.5)	2,006 (51.5)	.08
Likely	1,258 (37.9)	1,485 (42.4)	
Not sure/don’t know	386 (7.6)	188 (6.1)	

a All percentages were weighted with RDDWT_D sampling weight provided in National Immunization Survey–Teen, 2014.

b *P* values calculated by χ^2^.

c Includes multiracial and multiethnic.

d Based on the US Census poverty levels.

Among unvaccinated boys, variables independently predictive of intent to vaccinate against HPV were non-Hispanic black race/ethnicity (AOR, 1.89; 95% confidence interval [CI], 1.35–2.65) and Hispanic race/ethnicity (AOR, 1.87; 95% CI, 1.34–2.61) compared with non-Hispanic whites; mothers with less than a high school diploma (AOR, 2.41; 95% CI, 1.58–3.67) or high school diploma or general equivalency degree (AOR, 1.50; 95% CI, 1.12–2.02) compared with college graduates; never married mothers (AOR, 1.39; 95% CI, 1.06–1.81) compared with married mothers; and a provider recommendation for HPV vaccine (AOR, 1.87; 95% CI, 1.52–2.31) (Table 2). Among unvaccinated girls, predictors of HPV vaccination intention were Hispanic race/ethnicity (AOR, 1.57; 95% CI, 1.05–2.35) compared with non-Hispanic whites, mothers with less than a high school diploma (AOR, 1.86; 95% CI, 1.02–3.38) compared with college graduates, and a provider recommendation for the vaccine (AOR, 1.38; 95% CI, 1.05–1.82). Additionally, mothers with some college education were more likely to intend to vaccinate their sons (AOR, 1.21; 95% CI, 0.93–1.58) and less likely to intend to vaccinate their daughters (AOR, 0.69; 95% CI, 0.51–0.94) than were mothers with a college education.

**Table 2:** Association Between Characteristics of Adolescents Aged 13 to 17 Years and Intent to Receive Human Papillomavirus (HPV) Vaccine in the Next 12 Months, by Sex, National Immunization Survey–Teen, 2014^a^

Variable	Intends to Receive HPV Vaccine			Interaction *P* Value^b^
	Overall AOR (95% CI)	Boys AOR (95% CI)	Girls AOR (95% CI)	
**Sex**				
Boys	1 [Reference]	—		
Girls	1.06 (0.90–1.26)	—		
**Age, y**				
13	1 [Reference]	1 [Reference]	1 [Reference]	—
14	0.93 (0.72–1.19)	1.11 (0.80–1.53)	0.71 (0.50–1.02)	.07
15	0.89 (0.70–1.14)	0.83 (0.61–1.12)	1.01 (0.69–1.48)	.41
16	1.02 (0.79–1.32)	1.08 (0.77–1.51)	0.92 (0.63–1.33)	.52
17	0.74 (0.57–0.97)	0.83 (0.60–1.15)	0.64 (0.43–0.97)	.35
**Race/ethnicity**				
Non-Hispanic white	1 [Reference]	1 [Reference]	1 [Reference]	—
Non-Hispanic black	1.63 (1.24–2.15)	1.89 (1.35–2.65)	1.30 (0.83–2.04)	.19
Hispanic	1.69 (1.29–2.20)	1.87 (1.34–2.61)	1.57 (1.05–2.35)	.52
Non-Hispanic other^c^	1.27 (0.93–1.75)	1.18 (0.74–1.89)	1.42 (0.95–2.12)	.56
**Census region**				
South	1 [Reference]	1 [Reference]	1 [Reference]	—
Midwest	0.96 (0.80–1.15)	0.98 (0.77–1.23)	0.92 (0.68–1.23)	.74
Northeast	0.99 (0.80–1.23)	1.13 (0.86–1.48)	0.77 (0.55–1.08)	.08
West	0.99 (0.75–1.29)	1.02 (0.73–1.41)	0.92 (0.60–1.43)	.73
**Poverty status^d^**				
Above poverty	1 [Reference]	1 [Reference]	1 [Reference]	—
Below poverty	1.15 (0.88–1.50)	0.92 (0.67–1.26)	1.53 (1.00–2.35)	.06
**Mother’s education**				
Less than high school diploma	2.11 (1.48–2.99)	2.41 (1.58–3.67)	1.86 (1.02–3.38)	.49
High school diploma or general equivalency degree	1.25 (1.01–1.59)	1.50 (1.12–2.02)	0.97 (0.69–1.38)	.06
**Some college**	0.97 (0.79–1.19)	1.21 (0.93–1.58)	0.69 (0.51–0.94)	.006
College graduate	1 [Reference]	1 [Reference]	1 [Reference]	—
**Mother’s marital status**				
Married	1 [Reference]	1 [Reference]	1 [Reference]	—
Never married	1.37 (1.12–1.69)	1.39 (1.06–1.81)	1.33 (0.97–1.82)	.84
**Mother’s age, y**				
≤34	1 [Reference]	1 [Reference]	1 [Reference]	—
35–44	1.20 (0.88–1.63)	1.41 (0.93–2.13)	0.93 (0.58–1.49)	.20
≥45	1.22 (0.88–1.70)	1.44 (0.94–2.21)	0.99 (0.60–1.64)	.27
**Number of mother’s children aged <18 y**				
1	1 [Reference]	1 [Reference]	1 [Reference]	—
2 or 3	1.01 (0.84–1.23)	0.88 (0.70–1.11)	0.88 (0.70–1.11)	.06
≥4	0.63 (0.45–0.87)	0.74 (0.49–1.10)	0.50 (0.30–0.84)	.25
**Number of health care providers**				
1	1 [Reference]	1 [Reference]	1 [Reference]	—
2	1.15 (0.95–1.38)	1.08 (0.85–1.36)	1.24 (0.93–1.66)	.45
≥3	1.10 (0.88–1.38)	1.05 (0.79–1.39)	1.24 (0.88–1.74)	.45
**Health care provider recommended HPV vaccine**				
No	1 [Reference]	1 [Reference]	1 [Reference]	—
Yes	1.63 (1.38–1.93)	1.87 (1.52–2.31)	1.38 (1.05–1.82)	.08

Abbreviations: —, not applicable; AOR, adjusted odds ratio; CI, confidence interval.

a All percentages were weighted with RDDWT_D sampling weight provided in the National Immunization Survey–Teen, 2014. The models for both boys and girls were adjusted for all variables included in the table.

b *P* values calculated by logistic regression.

c Includes multiracial and multiethnic.

d Based on the US Census poverty levels.

Characteristics of adolescents whose parents reported that they were “not sure/don’t know” if they would vaccinate their children in the next 12 months are presented in Table 3. The largest percentage were boys (66.2%), non-Hispanic whites (46.8%), lived in the South (37.8%), lived above the poverty line (62.3%), had a college-graduate mother (30.5%), and had never received a recommendation for HPV vaccination from a health care provider (75.2%). Pearson χ^2^ tests indicated that, compared with those who expressed their intention about their likelihood to vaccinate their adolescents, parents who answered “not sure/don’t know” were significantly different based on adolescent’s race/ethnicity (*P* = .001), poverty status (*P* = .001), mother’s education (*P* = .003), and provider recommendation for HPV vaccine (*P* = .001).

**Table 3:** Characteristics of Adolescents Aged 13 to 17 Years and Multivariable Logistic Regression Predicting Parents’ Answering “Not Sure/Don’t Know” About Their Intention to Vaccinate Their Adolescents Against Human Papillomavirus (HPV) in the Next 12 Months, National Immunization Survey–Teen, 2014^a^

Variable	Known HPV Vaccine Intent (All Other Categories), n (Weighted %)	Unknown HPV Vaccine Intent (Not Sure/Don’t Know), n (Weighted %)	*P* Value^b^	Likelihood of Saying Not Sure/Don’t Know About HPV Vaccine Intention, AOR (95% CI)
**Overall**	8,992 (93.0)	574 (7.0)	—	—
**Adolescent’s sex**				
Female	3,491 (39.3)	188 (33.8)	.22	0.60 (0.42–0.87)
Male	5,501 (60.7)	386 (66.2)		1 [Reference]
**Adolescent’s age, y**				
13	2,102 (23.8)	133 (20.9)	.69	1 [Reference]
14	1,923 (21.1)	124 (19.6)		1.26 (0.71–2.25)
15	1,731 (18.3)	115 (21.4)		1.20 (0.65–2.22)
16	1,707 (20.0)	119 (22.8)		1.24 (0.66–2.34)
17	1,529 (16.8)	83 (15.3)		1.17 (0.61–2.25)
**Adolescent’s race/ethnicity**				
Non-Hispanic white	6,369 (61.8)	337 (46.8)	.001	1 [Reference]
Non-Hispanic black	718 (11.6)	70 (15.2)		1.90 (1.13–3.19)
Hispanic	1,097 (18.3)	77 (20.4)		1.60 (0.89–2.87)
Non-Hispanic other^c^	808 (9.0)	90 (17.6)		1.83 (1.11–3.02)
**Census region**				
South	3,249 (39.8)	225 (37.8)	.09	1 [Reference]
Midwest	2,102 (24.2)	102 (17.6)		1.07 (0.69–1.67)
Northeast	1,445 (14.60	124 (20.3)		1.56 (1.00–2.43)
West	2,196 (21.4)	123 (23.3)		1.01 (0.53–1.92)
**Poverty status^d^**				
Above poverty	7,503 (76.6)	409 (62.3)	.001	1 [Reference]
Below poverty	1,169 (19.4)	129 (30.5)		1.77 (1.07–2.92)
Unknown	320 (4.0)	36 (7.2)		—
**Mother’s education**				
Less than high school diploma	729 (10.2)	93 (18.5)	.003	1.61 (0.77–3.39)
High school diploma or general equivalency degree	1,524 (23.1)	122 (26.9)		1.21 (0.72–2.04)
Some college	2,591 (27.5)	160 (24.0)		1.26 (0.83–1.92)
College graduate	4,148 (39.2)	199 (30.5)		1 [Reference]
**Mother’s marital status**				
Married	6,918 (70.4)	411 (63.3)	.09	1 [Reference]
Never married	2,074 (29.6)	163 (36.6)		1.00 (0.66–1.52)
**Mother’s age, y**				
≤34	595 (7.2)	48 (11.0)	.11	1 [Reference]
35–44	3,655 (45.0)	245 (38.8)		0.83 (0.47–1.47)
≥45	4,742 (47.8)	281 (50.2)		1.26 (0.64–2.51)
**Number of mother’s children aged <18 y**				
1	3,513 (32.4)	234 (34.3)	.74	1 [Reference]
2 or 3	4,545 (54.2)	268 (51.1)		1.04 (0.69–1.59)
≥4	934 (13.3)	72 (14.6)		0.93 (0.51–1.72)
**Number of health care providers**				
1	4,559 (52.1)	326 (53.7)	.27	1 [Reference]
2	2,693 (28.9)	172 (32.4)		1.31 (0.87–1.96)
≥3	1,740 (19.0)	76 (13.8)		0.93 (0.57–1.51)
**Health care provider recommended HPV vaccine**				
No	5,227 (60.8)	426 (75.2)	.001	1 [Reference]
Yes	3,765 (39.2)	148 (24.8)		0.81 (0.54–1.21)

Abbreviations: —, not applicable; AOR, adjusted odds ratio; CI, confidence interval.

a All percentages were weighted with RDDWT_D sampling weight provided in the National Immunization Survey–Teen, 2014. The model was adjusted for all variables included in the table.

b *P* value of Pearson χ^2^ test indicating the difference in sociodemographic characteristics between respondents who answered “not sure/don’t know” versus all other categories of intent (ie, likely or unlikely to vaccinate) about their intention to vaccinate their adolescents in the next 12 months.

c Includes multiracial and multiethnic.

d Based on the US Census poverty levels.

Table 3 also presents multivariate logistic regression analysis of factors predicting a parental response of “not sure/don’t know” about intent to have their child vaccinated in the next 12 months. Parents of adolescent girls were less likely to say “not sure/don’t know” (AOR, 0.60; 95% CI, 0.42–0.87) than parents of boys. Furthermore, parents of non-Hispanic black adolescents (AOR, 1.90; 95% CI, 1.13–3.19) compared with parents of non-Hispanic whites and parents of adolescents living below the poverty level (AOR, 1.77; 95% CI, 1.07–2.92) were more likely to say “not sure/don’t know.” 

## Discussion

This secondary analysis of NIS-Teen 2014 data found that among parents of US adolescents aged 13 to 17 years, maternal education emerged as the strongest predictor of parental intent to obtain HPV vaccination for their children. Although provider recommendation was also a significant predictor of intent to vaccinate, the effect was not as strong as maternal education and was stronger for boys than girls. This differs from what is known about actual receipt of HPV vaccination, where provider recommendation is well established as the strongest predictor of actual vaccine uptake (20). This suggests that different factors may influence intent versus action, which should be acknowledged when considering interventions to elicit behavior change and progress across the stages of the Transtheoretical Model (15). For example, educational materials designed to increase awareness and understanding of HPV vaccination target people at the early stages of behavior change (ie, precontemplation and contemplation). Provider recommendation affects action — the initiation of HPV vaccination. However, our study indicate that additional factors, such as maternal education and race/ethnicity, may be important targets for transitioning to the preparation phase — those who intend to obtain HPV vaccination for their adolescents but have not yet had the first vaccination administered. Additionally, some targets for intervention may vary by the adolescent’s sex: mother’s marital status and non-Hispanic black race/ethnicity were significant predictors of intention to obtain HPV vaccination for parents of adolescent boys but not girls.

The lower intent to have their child vaccinated among college-educated mothers and parents of non-Hispanic white adolescents may be counterintuitive, because people from racial/ethnic minority groups and those with fewer economic resources often have challenges with access to medical care and uptake of medical services (21). However, these findings are consistent with previous research that found an association between the refusal of all childhood vaccines and white, college-educated mothers and high-income parents (22). Other studies also show higher rate of HPV vaccination among low-income and minority adolescents (19,23). A possible explanation for this phenomenon could be that daughters of above-poverty, non-Hispanic white parents have greater access to routine medical care, possibly leading to a lowered perceived need for HPV vaccine (21). Additionally, higher cervical cancer incidence and mortality among black and Hispanic minorities (3,24) may translate to a greater perceived susceptibility for HPV infection and a greater perceived need for preventive measures, such as HPV vaccination, than among non-Hispanic whites. A recent study supports this hypothesis, showing that maternal experiences with HPV-related disease have been associated with greater willingness to vaccinate their adolescents against HPV (25).

Our findings indicated some effect size differences among boys and girls in which parental intent to vaccinate was stronger for boys than for girls in the same category. For example, mothers with some college education were more likely to intend to vaccinate their sons and less likely to intend to vaccinate their daughters than mothers who were college graduates. Research suggests that parenting roles may be more protective of adolescent daughters than adolescent sons. This may be due in part to parents’ concerns about the unique risks for adolescent girls (eg, unwanted sexual involvement, pregnancy) (26). Several studies reported an opposition to vaccination of young girls, specifically for HPV, out of the unfounded fear that vaccination could have unwanted or negative influences on young girls’ sexual behavior (27,28). This attitude is fueled by 2 main theories: 1) that HPV vaccination may “ignite curiosity” leading to premature sexual activity because girls may believe that the vaccine will provide protection against all sexually transmitted infections (26) and 2) that parental consent for the HPV vaccine may imply parental approval of sexual activity (29). These ideas have been proven untrue in numerous studies; vaccination against HPV does not encourage risky sexual behavior and has actually been shown to support delayed sexual activity because of adolescents’ exposure to health information during the HPV vaccination (26). This coexistence of scientifically proven evidence and the continued presence and proliferation of incorrect ideas reinforces the need for provider involvement and recommendation of HPV vaccine. This approach can aid in overcoming any potential incorrect assumptions among parents of adolescents.

The importance of provider recommendation for HPV vaccination is consistent with earlier research that suggests this to be the strongest predictor of HPV vaccine receipt (20,23). Our study found that a large percentage (75.2%) of respondents answering “not sure/don’t know” about their intent to vaccinate their adolescents in the next 12 months had never had a health care provider recommend the vaccination. Clinicians are a trusted source and facilitator of vaccination behavior for all race/ethnicities (23). Thus, because such a large percentage of parents answering “not sure/don’t know” had not received a provider recommendation for the vaccine, it is not surprising that they remain on the fence about their intent to vaccinate their adolescents. Provider recommendation could be modified by interventions that target parental decision making along the stages of change continuum. Parents in the precontemplation and contemplation stages of HPV vaccination (such as those whose intent to vaccinate is “not sure/don’t know”) will need different tailored messages and discussions about the vaccine with clinicians. Current strategies to encourage providers to recommend HPV vaccination should be evaluated for effectiveness, and additional research is needed to better understand the quality of provider recommendation and how that differs by sociodemographic characteristics of adolescents. Additional research may also be needed to better understand the perceived barriers that clinicians report (30,31,32) to delivering HPV-related information and messages to patients at the different stages of decision making.

Approximately 7% of all survey respondents responded “not sure/don’t know” for whether they would vaccinate their adolescents for HPV in the next 12 months. Our analysis indicated that parents of adolescent boys and non-Hispanic blacks and parents living below the poverty line were more likely to remain undecided about their intention to have their children vaccinated. This may be attributed to the lower awareness of HPV and HPV vaccine among non-Hispanic black women and those living below the poverty line (33). While this is a small subset of the sample overall, it is important to consider that individuals who are in the precontemplation stage of decision making may benefit from different intervention strategies than those who are in the contemplation or preparation stages of change. Further research to examine attitudes toward HPV vaccine and beliefs of precontemplators is needed to inform development of interventions aimed at increasing HPV vaccine uptake.

A strength of this study is that it used a provider-verified vaccination history of a large, nationally representative sample of US adolescents. This reduces the potential for recall bias and social desirability bias with respect to parents reporting their child’s HPV vaccine status. However, this study also has a several limitations. Because of the cross-sectional nature of the data, causality cannot be determined, and temporal interpretations are limited. Second, although statistical adjustments were made to account for nonresponse and households without telephones, a possibility of nonresponse bias still exists. Our analysis was limited to the information included in NIS-Teen; therefore, we were not able to account for other factors associated with parental intention for HPV vaccination, such as personal or family history of gynecological cancer and sexually transmitted diseases, which have been shown to be positively associated with parental intention toward adolescents’ vaccination (34). Lastly, NIS-Teen does not conduct a follow-up survey to determine if those who intended to obtain HPV vaccination for their adolescents actually initiated and completed the vaccine series.

This study highlights several differences in parental intention toward HPV vaccination based on adolescents’ sociodemographic characteristics and the receipt of provider recommendation for HPV vaccination. Interventions should consider maternal education attainment, and studies should seek to understand why adolescents whose mothers have a college degree are not likely to intend to have their children vaccinated. Additionally, health care providers should actively engage in discussions with parents about HPV and strongly recommend the vaccine to all eligible patients concurrently with other routinely administered vaccinations to dispel any potential negative assumptions or opinions regarding HPV vaccination, especially among girls. Tailored efforts could be made to providers who serve different communities, primarily those who serve non-Hispanic whites, particularly those from high-income households or with mothers with college degrees. Additional studies are needed to examine provider–patient interactions in these settings to better understand how to effectively frame those efforts.
